# An analysis of six cases of acute intermittent porphyria (AIP)

**DOI:** 10.4103/0019-5545.31584

**Published:** 2006

**Authors:** Soumitra Ghosh, Pranit KR. Chaudhury, Hiranya K. Goswami

**Affiliations:** * Assistant Professor, Department of Psychiatry, Assam Medical College and Hospital, Dibrugarh, Assam; ** Professor and Head, Department of Psychiatry, Assam Medical College and Hospital, Dibrugarh, Assam; *** Professor and Head, Department of Psychiatry, Silchar Medical College, Silchar, Assam

**Keywords:** Acute intermittent porphyria, neuropsychiatric manifestation of AIP, mental symptoms of AIP

## Abstract

This analysis describes the diagnosis and psychiatric treatment modalities of 6 patients (5 women, 1 man; mean age 28.5 years) of acute intermittent porphyria (AIP), who presented to the Psychiatry OPD over a period of one year. The mean number of episodes was 2.83. Premorbid personality traits, clinical presentation, urine colour and urinary porphobilinogen titre were recorded. Among the 6 patients, 4 had abdominal pain, 5 had autonomic instability, all 6 had mental symptoms, 3 had depression, 2 came in delirium, and 3 had an episode of seizure.

## INTRODUCTION

Acute intermittent porphyria (AIP) is an autosomal dominant metabolic disorder that has various psychiatric manifestations. In AIP there is an abnormality in the haem biosynthetic pathway due to the deficiency of uroporphobilinogen I synthetase (porphobilinogen deaminase), which leads to excessive production of porphyrin precursors.^1^ The clinical features of AIP declare themselves at any stage from puberty onwards but mostly in the third decade of life. Penetrance of the gene responsible is incomplete, so that the disease often exists in a latent form and a family history may not be forthcoming.

The prevalence of AIP in the general adult population ranges between 1 and 8 per lakh.^1^,^2^ Tishler *et al.*^3^ reported the overall prevalence of AIP in the psychiatric population to be 210 per lakh. Clinical symptoms are rare before puberty and the diagnosis of AIP is suggested by a triad of symptoms (abdominal pain, neuropathy and mental changes) and porphobilinogen in the urine. Urinary porphobilinogen is a moderately sensitive test. The diagnosis is confirmed by demonstrating a decreasing level of porphobilinogen in the erythrocytes. Autonomic lability is also highly suggestive of the diagnosis.^4^

Mental disorders accompany attacks in 24% to 80% of cases and psychiatric symptoms can dominate the picture. Psychiatric manifestations include hysteria, anxiety, depression, phobias, psychosis, organic disorders, agitation, delirium and altered consciousness ranging from somnolence to coma,^5^ and the most common neuropsychiatric manifestation reported are delirium and depression.

Although the diagnosis of porphyria is seldom made, it is still underdiagnosed due to lack of suspicion about the condition and lack of adequate investigative facilities. Six cases of porphyria are described who presented to the Department of Psychiatry, Assam Medical College, Dibrugarh, Assam at different occasions over a period of one year from January 1997 to December 1997. One patient who presented with classical symptoms is described in detail.

## CASE 1

Ms YS, an 18-year-old tribal girl from a rural upper middle-class joint family with no history suggestive of psychiatric and metabolic disorders, had consulted many physicians and went to several psychiatric centres for 3 years since 1994. Her longitudinal history revealed that after attaining menarche at the age of 13 she used to have dysmenorrhoea, which subsided spontaneously. In 1994, at the age of 15 years, she was investigated for pain abdomen, and antitubercular treatment was started although there was no evidence of tuberculosis. The same treatment was continued by a physician on referral to Dibrugarh. She did not show any improvement; instead her condition deteriorated after discharge from hospital, because of which her medicines were discontinued. She however recovered spontaneously but continued to have episodes of pain abdomen, which made her very restless.

In March 1995, she was referred to a psychiatrist for vague somatic complaints. A diagnosis of conversion disorder was made. The patient never came for subsequent follow up, but on 26 December 1995 she reported with a history of poor sleep, crying spells, tingling of limbs, poor concentration and constipation. She was hospitalized and diagnosed as having ‘adjustment disorder with depressed mood’. Since her condition did not improve, she was taken to a private psychiatric nursing home at Guwahati on 24 February 1996, where EEG and CT scan of the brain were done and chlorpromazine and dothiapin was given at discharge. The patient was better for a month but again the same type of behaviour started and again she came to the Psychiatry OPD of Dibrugarh on 26 March 1996. She was admitted for her episodic abnormal behaviour, weeping spells, running away and suicidal attempt. Further, on the MSE she had obsessive rumination, pseudoauditory hallucination and depression. She was given clomipramine 175 mg, clonazepam 0.5 mg per day and 5 sessions of ECT. She recovered and remained well at home for 3 months with medicines although she still periodically suffered from abdominal pain.

In November 1996, while at home, she developed what appeared to be a generalized tonic–clonic seizure complicating with paralysis and semi-stupor.

In December 1996, she developed florid symptoms of psychosis and was treated at the Imphal Medical College. She was discharged as a case of schizophrenia and long-acting neuroleptics were prescribed.

She came back to the Psychiatry OPD, Dibrugarh, in February 1997, with suicidal thoughts, sleeplessness, restlessness, feeling of sadness, lack of interest, swelling of abdomen with colicky pain lasting for brief periods and amenorrhoea of 2 months. On MSE, the patient was irritable, depressed and help-seeking; affect was *la bella-type* and occasionally labile; an elementary type of auditory hallucination was found. No physical abnormality was found except sinus tachycardia. Her Hamilton Depressive Rating Score was 27. At that stage, clinical AIP was considered and urine was tested for porphobilinogen, which was present in 1:80 titre. An incidental finding at ultrasonography was the presence of gallbladder stone.

## CASE 2

Ms ND, a 35-year-old housewife and mother of two children, from a rural middle-class nuclear family, was referred on 16 August 1997 from the Medicine ward as a case of ‘hysteria’ for her uncooperative behaviour and slurred speech. She was admitted to the Medicine ward on 12 August 1997 in a semiconscious state after repeated attacks of generalized tonic–clonic seizures with no positive family history. Three months ago the patient had a similar seizure for which she was given phenytoin sodium, which caused marked confusion. Neurological assessment revealed the presence of peripheral neuropathy of all four limbs and residual XI and XII cranial nerve palsy as well as slurred speech. The patient was in delirium. Her high coloured urine raised a suspicion of porphyria. There was no pain abdomen. Urine showed porphobilinogen in 1:80 titre.

## CASE 3

Ms YC, a 16-year-old girl, was referred by a physician on 6 September 1997 from a rural area with no suggestive family history or past history of any psychiatric illness. She presented with complaints of sleeplessness, irritability, aggressive outburst since she was treated with chloroquin for her fever 20 days back. On examination, she was afebrile and complained of episodic pain abdomen. On physical examination, no abnormality was detected except paroxysmal tachycardia. But on MSE she had fearfulness and panic attack; urine test for porphobilinogen was done on suspicion and it was detected in 1:80 titre.

## CASE 4

Ms RB, a 34-year-old housewife and mother of two children, from an urban middle-class family, who was neurotic premorbidly and with no suggestive family history was referred to a psychiatrist in April 1996 by a general physician for her prolonged subjective unexplained multiple physical complaints including abdominal pain and constipation for the past 2 years. She was diagnosed as a case of somatoform disorder. During her several contacts with psychiatrists she had a fluctuating course that aggravated after imipramine, on her last visit in March 1997. She presented with bizarre somatic symptoms of delusional proportion with psychotic behaviour. Due to different presentation and for not showing improvements, porphyria was considered and urine for porphobilinogen was done and found positive in a 1:10 titre in consecutive samples. It was found negative during the remission period.

## CASE 5

Ms AC, a 38-year-old housewife and mother of three children, from an urban middle-class family, was stable premorbidly with no suggestive family history but short-lasting psychosis of 3 months which subsided spontaneously, was referred by a physician as a case of ‘hysteria’ to a psychiatrist with history of giddiness and loss of consciousness. On hospitalization the patient had atonic seizures and autonomic lability in the form of paroxysmal tachycardia and sweating and had anxiety with depression. Later, when she had severe pain abdomen with distension and vomiting, porphyria was considered and her consecutive urine samples were found positive for porphobilinogen in a 1:10 titre. On ultrasonography, cholecys-titis with cholelithiasis was detected.

## CASE 6

Mr DC, a 32-year-old married man, cultivator from a rural area with no suggestive family history, was admitted with a history of forgetfulness, weakness, poor food intake, sleeplessness, deterioration in social functioning, abnormal behaviour and generalized tonic–clonic seizures since one and a half months. He was never treated for seizures though these were present for 5 years. On starting with phenytoin, the patient gradually deteriorated and developed delirium and retention of urine. On noticing dark coloured urine, porphyria was suspected and porphobilinogen in urine was detected in 1:80 titre. Due to delayed diagnosis and treatment, the patient went into coma and died after 5 days.

## DISCUSSION

Table 1 shows the age and gender distribution of the 6 patients (5 women and 1 men; average mean age 28.5 years). AIP is more frequent in women between second and fourth decades of life.^6^,^7^ Average mean age of onset was 27.6 years. Mean number of episodes was 2.83. Premorbid personality did not bear any particular personality traits.

**Table 1:** Age and gender distribution

Case no.	Age (years)	Sex	Probable precipitating factor	Premorbid personality traits	Age of onset	No. of episodes
1. YS	18	F	Infection	Hysterical	15	7
2. ND	35	F	Poor calorie intake	Irritable, short tempered, mood swings	35	2
3. YC	16	F	Infection/chloroquin	Hysterical	16	1
4. RB	34	F	Not known	Neurotic	32	3
5. AC	38	F	Fluoxetin/amytryptaline	Stable	38	2
6. DC	32	M	Phenytoin	Introvert, stubborn	30	2

Table 2 shows their clinical presentation in detail including the urine colour and urinary porphobilinogen titre. Among 6 patients, 4 had abdominal pain, 5 autonomic instability, and mental symptoms in all 6. Three patients had depression and 2 came in delirium. Depression and delirium are two neuropsychiatric manifestations that are most commonly reported.^4^ Among the 6 patients, 3 had seizures. The life-time prevalence of AIP associated with seizure was 2.2% of all those with known AIP and 5.1% of all those with manifest AIP.^8^

**Table 2:** Clinical profile

Symptoms/signs	Case no. 1	Case no. 2	Case no. 3	Case no. 4	Case no. 5	Case no. 6	Total
Abdominal pain	+		+	+	+		4
Autonomic lability							
Peripheral neuropathy	+	+				+	3
Cranial nerve neuropathy		+					1
Arrythmia	+		+		+		3
Unstable blood pressure							0
Constipation	+	+		+	+	+	5
Vomiting					+		1
Seizure		+			+	+	3
Mental symptoms							
Delirium/confusion		+				+	2
Depression	+			+	+		3
Psychosis	+		+	+			3
Anxiety	+		+	+	+		4
High coloured urine	+	+	-	-	+	+	4
Urine for porphobilinigen (titre value)							
W and S test	1:80	1:80	1:80	1:10	1:30	1:80	

One patient (No. 6, DC) died while in coma due to AIP. According to Jeans *et al.*,^9^ mortality due to AIP was 3-fold compared to that in the general population during the past 50 years. The major cause of the increased mortality was the porphyric attack itself.

These patients were tested only qualitatively for porphobilinogen by using the Watson and Schwartz method. Quantitative tests for determination of 4-aminolevulinic acid (ALA) in urine could not be done due to lack of sophisticated laboratory facilities. But qualitative determination of porphobilinogen in the urine by Watson and Schwartz test is a simple and valuable screening test for the diagnosis of an acute attack of AIP. Hereditary coproporphyria (HCP) and variegated porphyria (VP) can be differentiated clinically from AIP by the presence of photosensitive cutaneous lesion and confirmed by titration of particular enzyme deficiency in erythrocytes, leukocytes and skin fibroblasts.

The Watson and Schwartz test is almost always positive during episodes of neuropsychiatric dysfunction but is positive only when the concentration of porphobilinogen in the urine is 3 to 5 times the upper limit of normal; as a consequence, both assays may be negative in latent cases and in patients in whom urinary excretion of porphobilinogen becomes normal following an acute attack. This however is by no means invariable and persistently elevated levels of porphobilinogen may occur. Caution is also required in certain febrile illness, in lead poisoning and in patients receiving phenothiazine drugs.^10^ Crimlisk^11^ reported that porphyria may be over-represented in psychiatric population, and negative screening test does not exclude the diagnosis. The diagnosis may be missed because it is not even considered or because of practical problems.

Depression is very common in porphyria but all textbooks are silent regarding safe antidepressants to be used. All standard textbooks mention imipramine and amitryptiline which can precipitate porphyria. There is uncertainty about newer antidepressants regarding their use in porphyria patients with depression, due to which the first patient could not be treated well. Clomipramine was given for a long time without signs of deterioration and unmodified ECT was safely used in one of the patients.

Again, regarding use of haloperidol and other anti-psychotic drugs besides phenothiazine nothing is mentioned in the textbooks. Theoretically we know that drugs that are metabolized by ‘cytochrome P450’ enzyme systems in the liver lead to the consumption of haem by cytochrome P450. This results in diminished levels of cellular concentration of haem, which in turn leads to depression of A-ALA synthetase with a corresponding increase in the rate of haem synthesis which may result in increased production of porphobilinogen in AIP patients.^12^

In conclusion, it is suggested that a list of safe antipsychotic and antidepressant drugs should be tried out and published to help in the treatment of porphyric patients with psychiatric manifestations.
